# Successful Fertility Preservation in a Case of Low-Grade Endometrial Stromal Sarcoma: A Multidisciplinary Approach Through the Oncofertility Network

**DOI:** 10.7759/cureus.74539

**Published:** 2024-11-26

**Authors:** Katsuhiro Ooi, Yu Wakimoto, Noriyoshi Oki, Mina Sakata, Seiji Mabuchi

**Affiliations:** 1 Gynecologic Oncology, Hyogo College of Medicine, Nishinomiya, JPN; 2 Obstetrics and Gynecology, Chibune General Hospital, Osaka, JPN; 3 Obstetrics and Gynecology, Osaka International Cancer Institute, Osaka, JPN

**Keywords:** fertility preservation, frozen-thawed embryo transfer, lgess, medroxyprogesterone acetate, progesterone receptor

## Abstract

Low-grade endometrial stromal sarcoma (LGESS) is a rare disease, accounting for less than 1% of all uterine malignancies. Standard treatment is total hysterectomy and bilateral tubal oophorectomy, although fertility preservation may be desirable because of the young age of onset. We document a case of fertility preservation in a 27-year-old nulligravida diagnosed with LGESS, which not only enabled the successful birth of two live infants but also underscores the efficacy of a multidisciplinary approach to patient treatment through the Hyogo Oncofertility Network (HOF-net). The patient underwent laparoscopic removal of a lesion initially suspected to be either a uterine myoma or an adnexal tumor. Through the HOF-net, a pioneering collaboration among patients, oncologists, and fertility specialists, she was connected to our department with the aim of achieving pregnancy in the future, even amidst her cancer diagnosis. Following the surgery, and during a medically advised period of contraception, assisted reproductive technology with the double stimulation (DuoStim) method was utilized to cryopreserve six embryos, followed by high-dose progestin therapy to mitigate the risk of cancer recurrence. Once the contraceptive mandate had been concluded, the implantation of the first thawed embryo during a hormone replacement cycle led to a viable pregnancy and the subsequent birth of a healthy child by cesarean section. Similarly, during the contraceptive period, after undergoing high-dose progestin therapy, a second thawed embryo transfer was performed, resulting in a successful pregnancy and the birth of a second child. Subsequently, she underwent a total hysterectomy and bilateral salpingectomy with preservation of both ovaries at another hospital 39 months after the initial surgery. No recurrence or residual disease was observed. The necessity for comprehensive informed consent was underscored by the potential for LGESS recurrence. Furthermore, the efficient coordination facilitated by the HOF-net enabled swift access to assisted reproductive services, aligning with the patient's primary healthcare plan. This case highlights the critical role of early engagement with assisted reproductive technologies and a multidisciplinary treatment strategy in facilitating successful outcomes for patients with LGESS, demonstrating the feasibility of fertility preservation in managing this condition.

## Introduction

Low-grade endometrial stromal sarcoma (LGESS), a rare subset of uterine malignancies, accounts for less than 1% of all cases [1]. The standard treatment protocol, which includes total hysterectomy and bilateral adnexectomy, invariably results in the irreversible loss of fertility [2]. This consequence is particularly poignant for younger women, who are predominantly affected by LGESS and for whom fertility preservation is often a critical concern. Additionally, the risk of LGESS recurrence further complicates the decision-making process regarding treatment options and fertility preservation. Given the recurrence risk associated with LGESS, the option of having a live birth prior to recurrence and then proceeding with the standard treatment protocol is highly desirable [1].

Here, we detail a case in which strategies for preserving fertility were successfully employed, allowing for the delivery of a live birth prior to recurrence and then proceeding with the standard treatment protocol. This approach culminated in the birth of two children shortly after the initial postoperative phase of a multidisciplinary treatment regimen through the Hyogo Oncofertility Network (HOF-net). This Oncofertility Network represents a collaborative system between oncologists and reproductive medicine specialists, aimed at providing information and treatment options related to oncofertility counseling for adolescent and young adult (AYA) patients diagnosed with cancer. Our approach notably included the application of embryo freezing after controlled ovarian stimulation using the double stimulation (DuoStim) method [3] during the contraceptive phase following enucleation. This case underscores the viability of fertility-sparing surgery (FSS) for LGESS patients and highlights the imperative of creating customized treatment plans. These plans should balance oncological safety with the potential for fertility outcomes, catering to the specific needs and preferences of the individual patient.

## Case presentation

**Figure 1:**
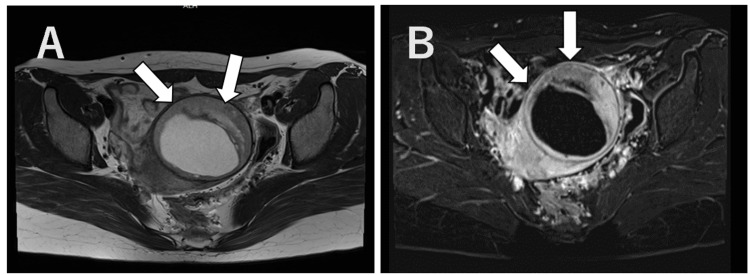
Contrast-enhanced MR images (A) Horizontal section T2. (B) Horizontal section T1. The arrow highlights an area with relatively weak contrast enhancement within the mass. This region exhibits a mildly high signal intensity on T2WI and a mildly low signal intensity on T1WI, suggesting it as the probable location of the malignant component. T2WI: T2-weighted imaging; T1WI: T1-weighted imaging

**Figure 2:**
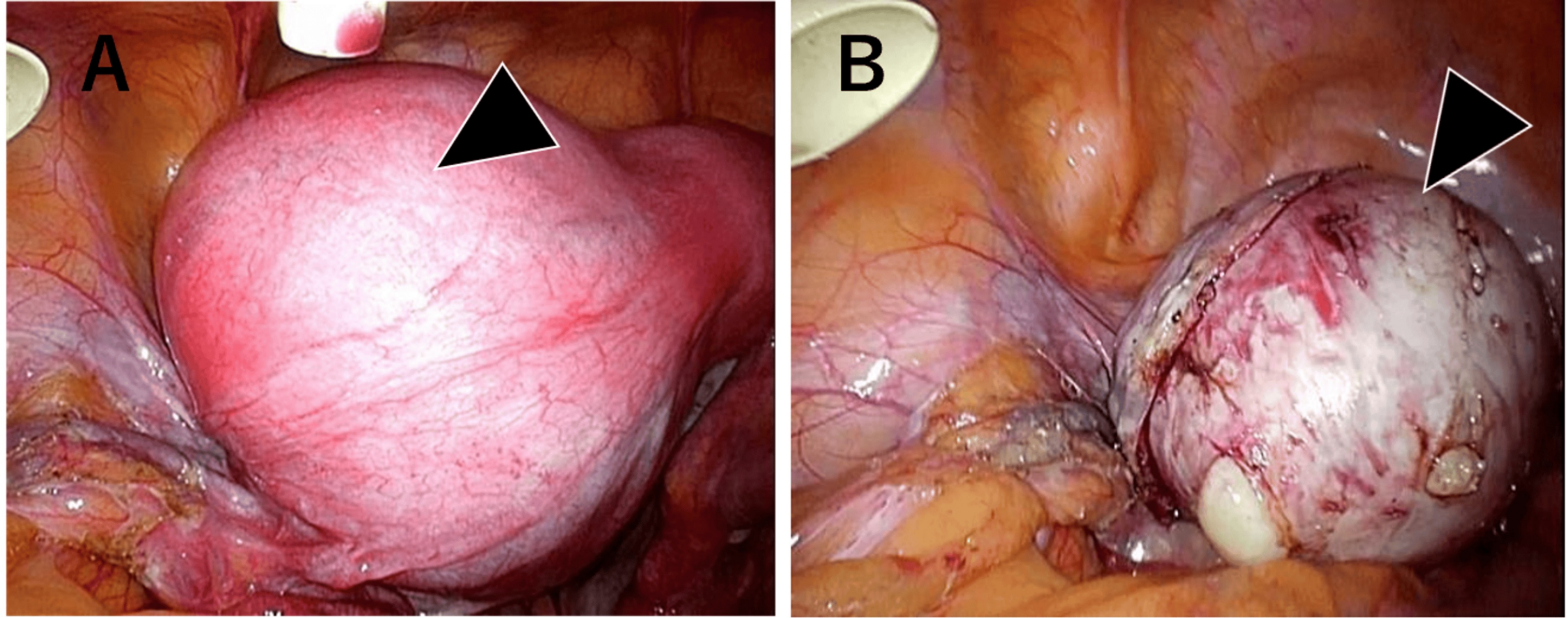
Laparoscopic surgery findings (A) Pre-enucleation imaging: A tumor, measuring approximately 10 cm in diameter, is located on the posterior wall of the uterine fundus. (B) Post-enucleation imaging: This image demonstrates the postoperative state following tumor enucleation. The tumor was successfully extracted through a small transverse incision in the lower abdomen, utilizing an enucleation bag for containment during laparoscopy. Although there was a partial leakage of the tumor contents during the procedure, there was no evidence of extensive dissemination or seeding of tumor cells.

**Figure 3:**
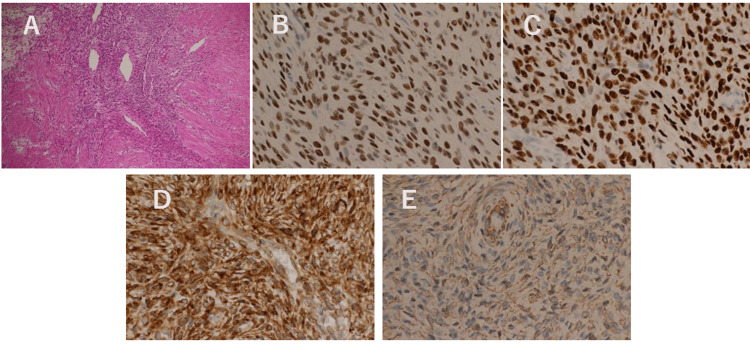
Microscopic and immunohistochemical analysis of the tissue obtained during the initial surgery (A) H&E staining. (B) ER. (C) PgR. (D) CD10. (E) αSMA. Immunohistochemistry showed ER (+), PgR (+), CD10 (+), αSMA (+), CD34 (-), h-caldesmon (-), and desmin (-). The image of the bundle-like proliferation of oval to short spindle-shaped cells is seen. There are small intervening vessels between cells. Vitrification and cystic voids were partially observed in the interstitium, with areas showing a tendency to invade the muscle layer. The number of fission images was 0-1/10 HPFs, and there were no coagulation necrosis images or lymphatic infiltrates.

The case involved a 27-year-old female patient with no antenatal or delivery history and no reported symptoms or medical history. Enhanced MRI conducted by a previous physician revealed a degenerated uterine myoma, characterized by a benign anterior segment showing mildly elevated signal intensity on T2-weighted imaging (T2WI), and a possibly malignant segment, due to its diminished contrast effect (Figure 1). An adnexal tumor or a degenerative uterine myoma was suspected, and laparoscopy-assisted enucleation was performed, resulting in the removal of a 10 cm, 115 g mass with clear boundaries, multiple internal foci, and a yellowish liquid content, indicative of uterine origin (Figure 2). Postoperative histopathology confirmed a diagnosis of LGESS (Figure 3), stage IB (T1bN0M0). Concerns about a risk of recurrence led to the recommendation of a total hysterectomy and bilateral adnexectomy by the patient's primary physician. However, desiring to conceive, the patient was referred to our department through the HOF-net to plan for an early pregnancy.

In the contraceptive phase post-enucleation, embryo cryopreservation was achieved by retrieving 12 oocytes on the 21st postoperative day via ovarian stimulation using a random start protocol and two more oocytes on the 28th day using the DuoStim method, resulting in six frozen embryos. Following 50-84 days of high-dose progestin treatment post-surgery, PET/CT and MRI confirmed no residual tumor. Four months postoperatively, a successful pregnancy was achieved on the first attempt through a frozen-thawed embryo transfer within a hormone replacement cycle, with no perinatal issues for the mother or child. At 37 weeks and two days of gestation, 12 months post-surgery, a healthy boy weighing 2950 g with an Apgar score of 8/9 was delivered via a planned cesarean section, with no signs of recurrence observed during the procedure.

Seeking a second pregnancy, the patient returned to our department 23 months post-surgery after six months of medroxyprogesterone acetate (MPA) therapy. With no evidence of metastasis or recurrence from contrast-enhanced CT, another frozen embryo transfer was conducted. The second pregnancy was established, progressing without complications, and resulted in the birth of a boy weighing 2920 g, via planned cesarean section at 37 weeks gestation, 34 months after the initial surgery. The baby scored 8/9 on the Apgar scale.

Following her second childbirth, the patient was in good health up to the one-month postpartum visit. At her request, and after consultation, she underwent a total hysterectomy and bilateral salpingectomy with preservation of both ovaries at another hospital 39 months after the initial surgery. No recurrence or residual disease was observed.

## Discussion

In this case, the patient was diagnosed with LGESS and underwent fertility-sparing surgery and embryo cryopreservation, followed by two successful childbirths and a total hysterectomy within 39 months. Among patients diagnosed with LGESS, this case is notable for the patient's achievement of two successful pregnancies and the subsequent hysterectomy, which is the standard procedure for LGESS, with no recurrence. This remarkable outcome was facilitated by leveraging the Oncofertility Network alongside the integration of assisted reproductive technologies within a comprehensive treatment strategy. Notably, the Gifu Prefecture Oncofertility Network, established in 2013, marked the inception of such regional medical collaboration in Japan. Our department replicated a similar network in Hyogo Prefecture three years later, fostering prompt and seamless cooperation among cancer treatment specialists [4]. The utilization of the Oncofertility Network in this instance expedited the deployment of assisted reproductive technologies immediately after tumor debulking. During the post-debulking contraceptive period, ovarian stimulation was executed twice within the same ovarian cycle, during both the follicular and luteal phases, by means of the DuoStim method, thereby maximizing embryo yield in a constrained timeframe. This method is recommended for those seeking fertility preservation prior to cancer treatment and for older patients with a diminished ovarian response [3]. This strategy significantly increased the count of high-quality blastocysts, thus enhancing the possibility of early pregnancy success upon transplantation. In the context of infertility treatment aimed at producing two infants, the first embryo transfer led to pregnancy on both occasions.

The median onset age for LGESS is 41.8 years, with 35.7% of cases occurring among women aged 18-44 and 14.4% being nulliparous [5,6]. Given the relatively positive prognosis, with a five-year survival rate of 98% and a 10-year survival rate of 89% for stage I disease [5,6], the pursuit of fertility-preserving treatments is warranted. In this case, the decision to preserve fertility was supported by clearly defined tumor margins conducive to complete resection, a mitotic count of 0-1/10 high-power fields (HPF), and an absence of coagulative tumor necrosis or lymphovascular invasion, indicating a minimal risk of recurrence [7]. Recurrence has been linked to a mitotic count exceeding 3/10 HPF, coagulative tumor necrosis, and lymphovascular invasion [7]. Considering the recurrence rates documented in stages IA (0/6, no recurrence) versus IB (10/11, 90.9% recurrence) [5,7], opting for a total hysterectomy post-childbearing is advisable [5,6].

Literature on fertility preservation in LGESS is limited, and while the risk of recurrence remains, undergoing such treatments after the fulfillment of fertility desires is generally recommended [1]. Discussion of prognosis and recurrence risk post-uterus preservation and pregnancy achievement in LGESS cases is predominantly confined to case reports and small series. Laurelli et al. reported that among six LGESS-diagnosed patients, two conceived successfully without complications, one endured a miscarriage, but no recurrences were observed within a two-year follow-up [1]. Jin et al. detailed five cases of conservative LGESS treatment, where patients underwent uterine lesion excision and received 160-320 mg/day of megestrol acetate for six months. Three patients conceived within a 21-55-month follow-up, with one experiencing recurrence during treatment, necessitating a hysterectomy [8]. Zheng et al. most recently reported on five LGESS patients who had undergone conservative surgery and a year of hormone therapy, with four suffering recurrences in the uterus and iliac blood vessels. The median no-recurrence survival span was 38 months, with two patients conceiving post-salvage surgery without complications [2].

Estrogen and progesterone receptor expression is deemed a favorable prognostic factor in LGESS, with hormone therapy proving effective when both receptors are present. During the contraception period, hormone therapy with MPA was administered to reduce the risk of recurrence. MPA, the most commonly utilized progestin, is believed to mediate antitumor effects via the progesterone receptor (PgR) [9]. Aromatase inhibitors, such as letrozole, which curtail aromatase activity and diminish estrogen production, are deemed effective in recurrence prevention in LGESS [10]. The National Comprehensive Cancer Network and the European Society of Gynaecological Oncology recognize hormone therapy as a pivotal component of LGESS management.

The preoperative diagnosis of LGESS is challenging, reflecting the disease's rarity, constituting less than 1% of uterine malignancies. Furthermore, nonspecific clinical and imaging manifestations often lead to it being misdiagnosed as a benign condition such as uterine myoma, adenomyosis, or polyps [8]. Furthermore, despite LGESS's characteristic vascular invasion of the myometrium, assessing tumor invasion while preserving the uterus is challenging, necessitating excision and histopathological examination for accurate diagnosis [8].

## Conclusions

Our case report contributes to the growing body of literature advocating for a personalized, multidisciplinary approach to cancer treatment that incorporates fertility preservation. The establishment and utilization of networks like the Oncofertility Network are pivotal in facilitating these comprehensive care models, ensuring that patients do not have to choose between cancer treatment and the possibility of future parenthood.

## References

[1] Laufer J, Scasso S, Kim B, Shahi M, Mariani A (2023). Fertility-sparing management of low-grade endometrial stromal sarcoma. Int J Gynecol Cancer.

[2] Zheng Y, Yin Q, Yang X, Dong R (2020). Fertility-sparing management of low-grade endometrial stromal sarcoma: analysis of an institutional series, a population-based analysis and review of the literature. Ann Transl Med.

[3] Vaiarelli A, Cimadomo D, Petriglia C (2020). DuoStim - a reproducible strategy to obtain more oocytes and competent embryos in a short time-frame aimed at fertility preservation and IVF purposes. A systematic review. Ups J Med Sci.

[4] Takai Y (2018). Recent advances in oncofertility care worldwide and in Japan. Reprod Med Biol.

[5] Bai H, Yang J, Cao D (2014). Ovary and uterus-sparing procedures for low-grade endometrial stromal sarcoma: a retrospective study of 153 cases. Gynecol Oncol.

[6] Xie W, Cao D, Yang J (2016). Fertility-sparing surgery for patients with low-grade endometrial stromal sarcoma. Oncotarget.

[7] Borella F, Bertero L, Cassoni P (2022). Low-grade uterine endometrial stromal sarcoma: prognostic analysis of clinico-pathological characteristics, surgical management, and adjuvant treatments. Experience from two referral centers. Front Oncol.

[8] Jin Y, Li Y, Deng CY, Tian QJ, Chen H, Pan LY (2015). Fertility-sparing treatment of low-grade endometrial stromal sarcoma. Int J Clin Exp Med.

[9] Sasano H, Harada N (1998). Intratumoral aromatase in human breast, endometrial, and ovarian malignancies. Endocr Rev.

[10] Reich O, Regauer S (2004). Aromatase expression in low-grade endometrial stromal sarcomas: an immunohistochemical study. Mod Pathol.

